# Beneficial Results Following, and Apparently Depending upon, the Use of Teucrium Marum in Aural Polypus

**Published:** 1872-08-01

**Authors:** John Bartlett


### BENEFICIAL RESULTS FOLLOWING, AND APPARENTLY DEPENDING UPON, THE USE OF TEUCRIUM MARUM IN AURAL POLYPUS.

BY JOHN BARTLETT, M. D.

After having been troubled with otorrhœa for a year or more, in December last I became quite deaf. Upon applying to Prof. E. L. Holmes of Chicago, in March, a soft polypus, apparently springing from the membrana tympani was discovered, occluding the meatus. The shape and location of the growth was such that the aurist advised that its removal be deferred for a month or more, and that meanwhile its destruction might be attempted by local applications. Being familiar with the unsatisfactory results of the treatment of these polypi with caustics, and of the possibility of extraction proving, in the end, only a palliative measure, I cast about for some other means of relieving myself from this growth, which, on account of chronic deafness in the other ear, was seriously interfering with my comfort and business.

Being aware that teucrium marum had been used by Mayer as a local application, with good results in nasal polypus, and that it was recommended with confidence as an internal remedy for nasal and aural polypi by homœopathic writers, I determined to try it. Accordingly I took of the tincture one-half a minim four times daily. In one week Dr. D. B. Hillis of Keokuk, who was kind enough to overlook the treatment, discovered a slight contraction of the growth. In another week its dimensions were so far diminished that, immediately after syringing the secretion from the ear, an air space alongside of the polypus permitted for some minutes quite satisfactory hearing. The growth continued to shrink slowly and at the end of a month the passage to the drum which at first rapidly refilled with secretion remained permanently clear. The watch could be heard at six inches, and the stethoscope used with some confidence. I remarked that when the medicine was discontinued, as when traveling, or because of an occasional slight dizziness which I thought might be attributed to its action in diminishing the secretion from the ear, deafness returned. Upon resorting to the remedy again, hearing improved. During the first weeks of the treatment the teucrium was applied locally by dropping into the ear after syringing five or six drops of the tincture, diluted four times with water.

In June I called again upon Dr. Holmes; preparatory to an examination, he syringed the ear, and in so doing detached the polypus. Hearing was instantly restored. Some hemorrhage followed the detachment. The secretion, which had been copious, stopped within three days, and the meatus seemed in a better condition than for several years past. Upon careful inspection of the ear three weeks after the removal of the polypus, no evidence of a reproduction of the growth was discovered, the use of the teucrium, meanwhile, having been virtually abandoned.

It chanced that at the time of my first using the teucrium, I had under observation two cases of aural polypus, both of long standing; one had been detected seven years, and the other twelve years previously. These patients had declined active measures for relief. I sought them out and prescribed the tincture for them.

In the first case the polypus, of the mulberry variety, arising in the middle ear, reached outwardly so far as to have been within the touch of the finger. In six weeks, under the use of teucrium, its volume had diminished four-fifths, and it had receded within the middle ear. No uneasiness was experienced from the use of the medicine. During the latter half of the treatment, it had been used locally.

In the second case, no examination had been made recently. At the last observation the tympanal cavity was filled with two soft polypi. At the time of administering the teucrium, the deafness in the diseased ear was decided. For seven months the patient had not been able to hear the rumbling of wagons over the paved street. Ten days after taking the first dose of teucrium he called for more, stating that his hearing had much improved; he could hear ordinary conversation, and the ticking of a clock was audible at a distance of thirty feet.

The first patient has been lost sight of for the present; the second discontinued the medicine, until recently, because, as he believed, it produced headache.

The botanical history of teucrium marum, kindly furnished by Prof. H. H. Babcock, is subjoined:

"*Teucrium Marum*, W., or cat thyme, is an evergreen shrub, eighteen inches high, a native of Spain, whence it was introduced into England in 1640. It blooms in July or August, producing pale, purple flowers. The recent leaves, when crushed, emit an aromatic odor, causing sneezing, but are bitter to the taste, whence the specific name from the Arabic, *Mar*, bitter. Cats are very fond of the plants, eating it with avidity. About fifty species of *Teucrium* are known, one only native of America, the *T. Comadeuse*, L., common throughout the United States, abundant near Chicago, and now in bloom."

The following account of the medicinal properties and uses of teucrium is taken from that admirable pharmacologia, Lessing's *Heilmittel Lehre*, Ed. 1844. It comprises all the information concerning the properties of the article which has fallen under my notice:

"Teucrium Marum is an unjustly neglected remedy, which by the older physicians was held in great repute as a fine volatile stimulating and anti-spasmodic nervine. It especially restores tone to the sensory ganglia, its salutary effects seeming to be specifically directed to the sensorium proper, so that while other articles of its class cause slight headache, this alone removes cephalalgia, when of a purely nervous character. The older physicians gave it in diseases of the respiratory organs; in spasmodic asthma; in difficult expectoration; in tracheal croup, and even in dropsical affections of the chest accompanied by serious nervous complications. They also used it in milder forms of typhus fever, in syncope, and nervous apoplexy. It is now for the most part limited to external use, and particularly to the treatment of diseased formations of the nasal mucous membrane, and polypi. In such cases it has been used as a sternutatory by Mayer and Kleeman with the best results."

The upper flowering stems and the leaves are used.

Dose, and mode of administration: Internally, seldom, one scruple to one drachm, in powder.

An infusion may be made in the proportion of half an ounce to an ounce to six or eight ounces of water. And as a decoction, three drachms to three cupsful of water.

Externally, as a sternutatory, a pinch of the powder may be taken from three to five times a day.

The doses of the infusion and decoction are not given, they may be deduced from that of the powder.

The tincture used by myself was made by macerating one part of the plant in ten of proof spirits.

